# Genetic association and functional analysis of rs7903456 in *FAM35A* gene and hyperuricemia: a population based study

**DOI:** 10.1038/s41598-018-27956-3

**Published:** 2018-06-25

**Authors:** Feng Yan, Peng Sun, Huishou Zhao, Changhai Zhao, Nana Zhang, Yujie Dai

**Affiliations:** 1 Department of Clinical Nutrition, Xijing Hospital, the Fourth Military Medical University, Xi’an, Shaanxi China; 2 Department of Neurosurgery, Tangdu Hospital, the Fourth Military Medical University, Xi’an, Shaanxi China; 3 Department of Cardiology, Xijing Hospital, the Fourth Military Medical University, Xi’an, Shaanxi China; 4 Department of Endocrinology, Xijing Hospital, the Fourth Military Medical University, Xi’an, Shaanxi China

## Abstract

Recent studies have identified SNP rs7903456 of *FAM35A* to be associated with gout. Because of the close connections between hyperuricemia and gout, we hypothesized that the effect of rs7903456 on gout might be mediated by hyperuricemia or its related quantitative trait, uric acid level. We investigated the association between 31 SNPs of *FAM35A*, including rs7903456, and hyperuricemia based on 2,773 hyperuricemia patients and controls. We fitted a simple model for each of these 31 SNPs to screen the candidate SNP for further analyses. Moreover, we selected potential confounders and fitted a multivariate model to investigate the adjusted effects of the targeted SNPs. Both disease status of hyperuricemia and blood uric acid level were considered as the main phenotype. We have identified rs7903456 to be associated with hyperuricemia and uric acid level. The significant signal was identified between rs7903456 and uric acid level after adjusted by several potential confounders. Our findings showed that the T allele of rs7903456 could increase the uric acid level by ~10 mmol/L on average after adjusting several biochemical and clinical variables. Our findings indicated that the previously identified effects of rs7903456 on gout might partly be mediated by its effect on uric acid levels.

## Introduction

Hyperuricemia is an abnormally increased level of uric acid in the blood. It is a major cause of gout^1–3^ and is also related to multiple metabolism-related disorders, including hypertension, obesity, and hyperinsulinemia^4–6^. Previous studies have identified that the heritability of blood uric acid levels is no less than 30%, which indicates that genetics have played a key role on the distribution of this quantitative trait^7^. A genome-wide linkage study has identified several novel loci linked to uric acid, although replications are weak^8^. In addition, a candidate gene-based association study has identified *HNF4G*, *β3-AR*, and *ABCG2* to be significantly associated with hyperuricemia in Chinese and Korean populations^9–11^. Despite these early studies, the genetic etiology of hyperuricemia is still unclear, and additional studies are needed in the future.

A recent study conducted by Nakayama *et al*. revealed several significant single nucleotide polymorphisms (SNP), but especially rs7903456 of *FAM35A*, to be associated with gout^12^. Because of the close connections between hyperuricemia and gout, we hypothesized that the effect of rs7903456 on gout might be mediated by hyperuricemia or its related quantitative trait, uric acid level. So far, although Nakayama *et al*. reported that some genes near *FAM35A* including *GLUD1* have some possible relationship with gout^12^, the function of *FAM35A* is still totally unknown. Thus, it might be interesting to investigate the target loci of rs7903456 through analysis based on bioinformatics tools.

In this study, we investigated the potential association between 31 preselected SNPs of *FAM35A* including rs7903456 and hyperuricemia based on approximately 2,773 hyperuricemia patients and controls. In addition to the major phenotypes, we also collected several clinical variables that might potentially confound the association signals. Both simple and multivariate models were fitted to identify the underlying independent effects of SNPs on the disease status of hyperuricemia and uric acid level.

## Materials and Methods

### Subjects

In our study, a total of 981 hyperuricemic individuals without gout and 1,792 normouricemic controls were recruited from Xijing Hospital, the Fourth Military Medical University between 2013 and 2016. The study protocol was approved by the Ethics Committee of Xijing Hospital of the Fourth Military Medical University in accordance with the ethical guidelines of the Helsinki Declaration of 1975 (revised in 2008). Written informed consent was obtained from participants before the start of the study. Hyperuricemia was defined as serum uric acid concentrations ≥416 μmol/L (male) or ≥357 μmol/L (female). Normouricemia was defined as serum uric acid <416 μmol/L (male) or <357 μmol/L (female). The controls were enrolled based on the selection criteria of frequency-matched age (±5 years) and gender of the patients. The age of enrolled subjects was between 25 and 60 years, and the body mass index (BMI) ranged from 21 to 28 kg/m^2^. All enrolled subjects had serum blood urea nitrogen and serum creatinine within the normal range. Subjects with diabetes, hypertension, congestive heart failure, kidney or liver diseases, as well as alcohol or tobacco addiction, were excluded, and individuals with a history of serum uric acid lowering agents were also excluded. Serum biochemical tests of all subjects were conducted, and biochemical parameters including total cholesterol, triglycerides, low density lipoprotein cholesterol, high density lipoprotein cholesterol, fasting glucose, creatinine, urea nitrogen, and uric acid in the plasma were measured or recorded.

### SNPs selection and Genotyping

We searched for all SNPs with minor allele frequencies (MAF) ≥0.01 within the region of the *FAM35A* gene in the 1000 Genomes Chinese Han Beijing population (CHB). Then, MAF ≥ 0.01 with pair-wise tagging and r^2^ ≥ 0.8 were used as the cut-off criteria during tag SNP selection, which generated 31 tag SNPs covering the region of the *FAM35A* gene for our study. Basic information on the 31 selected SNPs is summarized in the Supplemental Table S1. As we can see from this table, most of our selected SNPs are located at the intronic region. All of our selected SNPs had *P* values greater than 0.05 on the Hardy-Weinberg equilibrium test. Genomic DNA was extracted from peripheral blood leukocytes according to the manufacturer’s protocol (Genomic DNA kit, Axygen Scientific Inc., California, USA). Genotyping was performed for all SNPs using the Sequenom Mass ARRAY RS1000 system (Sequenom, San Diego, California, USA). The results were processed using Typer Analyzer software, and genotype data were generated from the samples. Case and control statuses were blinded during all genotyping processes for quality control. Five percent of the random samples were repeated, and the results were 100% concordant.

### Statistical and Bioinformatics analyses

Clinical variables and age were compared between hyperuricemia patients and the controls using a two-sample t-test. The distribution of gender between the two groups was examined by χ^2^ test. Genetic association analyses were conducted by Plink^13^. Since multiple clinical variables have shown significant differences between our hyperuricemic patients and controls, it was very important to adjust for some of these variables to remove the potential confounding effects. To achieve this goal, we implemented a three-step strategy as follows: 1) We fitted a simple model for each of these 31 selected SNPs with only age and gender adjustments, and significant SNPs will be obtained and kept for further analysis. 2) We took those clinical variables one-by-one as phenotypes and fitted linear models with the significant SNPs obtained from the previous step (including age and gender as covariates) to identify the potential confounders. Significant clinical variables will be kept for further analysis. 3) A multivariate model will be fitted with age, gender and the confounders identified from step 2 to investigate the adjusted effects of the targeted SNPs. Both disease status of hyperuricemia and blood uric acid level were considered as the main phenotype. General statistical tests were performed by R software^14^. *P* = 0.05 was considered as the significance threshold for single statistical tests. Bonferroni corrections were applied as necessary to address multiple comparisons. To examine the potential functional significance of the identified SNPs, we utilized GTEx to investigate the eQTL pattern of the significant SNPs^15^. Furthermore, we performed power calculations by using PGA v2.0^16^. Our sample size can detect SNP association with 88% power, at a false positive rate of 5% and a presumed odds ratio (OR) of 1.5.

## Results

### Simple model fitting

Demographic and clinical characteristics of hyperuricemic patients and controls were shown in Table 1. All of the clinical variables tested in our study subjects have shown significant differences between patients and controls. The simple models for the disease status of hyperuricemia and uric acid level were fitted. The full results are summarized in Table 2. A total of 31 SNPs were tested, and the *P* value threshold used here was 0.05/31 ≈ 0.0016. With this threshold, we have identified SNP rs7903456 to be significantly associated with the disease status of hyperuricemia (OR = 1.33, *P* = 3.54 × 10^−6^) after adjustment by age and gender, and this significant signal can be replicated by analysis of the blood level of uric acid (*β* = 10.85, *P* = 6.82 × 10^−5^). The Q-Q plot of the full results of the simple model was shown in Supplemental Fig. S1.

**Table 1:** Demographic and clinical characteristics of hyperuricemic patients and normouricemic controls.

Characteristics	Male		*P* value	Female		*P* value
	Patients	Controls		Patients	Controls	
Number	770	1,408	NA	211	384	NA
Age (years), mean ± SD	44.73 ± 8.55	44.81 ± 8.45	0.8327	46.24 ± 8.63	46.13 ± 8.04	0.8802
BMI (kg/m^2^)	25.5 ± 1.27	23.6 ± 0.88	<0.001	23.7 ± 0.75	22.8 ± 0.58	<0.001
Total cholesterol (mmol/L)	4.18 ± 0.57	3.91 ± 0.53	<0.001	4.22 ± 0.54	3.93 ± 0.57	<0.001
Triglycerides (mmol/L)	2.36 ± 0.42	1.79 ± 0.30	<0.001	1.84 ± 0.28	1.57 ± 0.33	<0.001
LDL-C (mmol/L)	2.44 ± 0.28	2.21 ± 0.29	<0.001	2.47 ± 0.30	2.26 ± 0.33	<0.001
HDL-C (mmol/L)	1.11 ± 0.18	1.15 ± 0.19	<0.001	1.17 ± 0.17	1.19 ± 0.20	0.2041
Fasting glucose (mmol/L)	5.21 ± 0.47	4.78 ± 0.44	<0.001	5.18 ± 0.42	4.66 ± 0.41	<0.001
Creatinine (μmol/L)	91.42 ± 7.95	77.68 ± 8.24	<0.001	72.75 ± 7.60	67.21 ± 7.33	<0.001
Urea nitrogen (mmol/L)	5.21 ± 0.69	4.73 ± 0.68	<0.001	4.69 ± 0.63	4.18 ± 0.60	<0.001
Uric acid (μmol/L)	462.84 ± 44.20	404.52 ± 27.15	<0.001	298.52 ± 52.22	234.52 ± 35.85	<0.001

SD: standard deviation; BMI, body mass index; LDL-C, low density lipoprotein cholesterol; HDL-C, high density lipoprotein cholesterol; NA, not applicable.

**Table 2:** Results of genetic association analyses with hyperuricemia disease status and uric acid level using the simple model.

CHR	MARKER	POSITION	A1	MAF	OR	*P*_logistic_	BETA	*P*_UA_
10	rs7903456*	87159562	T	0.29	1.33	3.54E-06	10.85	6.82E-05
10	rs377405832	87151317	G	0.02	1.31	0.15	17.49	0.04
10	rs187235859	87162364	A	0.07	1.10	0.36	7.11	0.13
10	rs185147308	87177555	T	0.03	1.15	0.39	9.29	0.20
10	rs181570058	87103090	A	0.03	1.14	0.41	−2.87	0.69
10	rs375246821	87140690	G	0.02	0.85	0.42	−2.54	0.77
10	rs544765887	87151309	C	0.02	0.85	0.43	−9.00	0.30
10	rs116955887	87158872	G	0.09	1.08	0.43	6.41	0.13
10	rs189796790	87175350	T	0.02	0.87	0.45	−4.65	0.57
10	rs10466230	87147698	C	0.04	0.91	0.48	−8.05	0.18
10	rs200718310	87157420	T	0.07	1.07	0.54	6.23	0.20
10	rs11202365	87170492	T	0.21	1.04	0.59	1.50	0.62
10	rs77534648	87180141	T	0.08	1.06	0.59	1.06	0.82
10	rs138542761	87151670	C	0.06	1.06	0.65	3.34	0.52
10	rs191015049	87166777	A	0.07	0.95	0.66	2.13	0.67
10	rs200289839	87129688	A	0.11	0.96	0.66	−0.72	0.86
10	rs368106548	87170893	A	0.04	1.06	0.69	1.66	0.79
10	rs190379103	87179590	T	0.04	0.94	0.71	−4.20	0.53
10	rs151327238	87156665	G	0.05	1.05	0.72	−0.47	0.94
10	rs55892577	87169165	C	0.10	0.97	0.72	0.18	0.97
10	rs67141022	87096385	C	0.10	1.03	0.76	−0.88	0.83
10	rs117800846	87171219	A	0.07	1.03	0.76	2.43	0.62
10	rs7089418	87126743	C	0.12	0.97	0.77	0.10	0.98
10	rs148634296	87113822	G	0.26	1.02	0.78	3.08	0.27
10	rs186029096	87120536	T	0.06	0.97	0.80	−1.86	0.71
10	rs4933432	87165772	A	0.39	0.99	0.83	2.06	0.42
10	rs1885931	87147082	A	0.39	0.99	0.83	0.02	0.99
10	rs575094327	87154671	T	0.04	1.03	0.85	−2.14	0.73
10	rs374457039	87167593	G	0.06	1.02	0.87	4.10	0.43
10	rs533233110	87168299	G	0.05	1.02	0.90	4.51	0.43
10	rs550312128	87164678	T	0.07	1.01	0.94	3.93	0.40

CHR, chromosome; A1, effect allele; OR, odd ratio.

*P*_logistic_, *P* values of logistic regression using disease status as the phenotype; *P*_UA_, *P* values of the linear regression using blood uric acid level as the phenotype. Significant results are highlighted in bold.

^*^The 95% CI of the rs7903456 is 1.18–1.49.

### Confounding factors and multivariate model fitting

To identify the potential confounding factors that need to be adjusted in the multivariate model, we have fitted several simple models to investigate the potential relationships between SNP rs7903456 and the clinical variables tested in our study subjects. The results are summarized in Supplemental Fig. S2. A total of 6 out of the 8 clinical variables, including BMI (body mass index), fasting glucose level, total cholesterol level, triglyceride level, urea nitrogen level and creatinine level, were identified to be significant and therefore need to be adjusted in the multivariate model. HDL (high density lipoprotein cholesterol) and LDL (low density lipoprotein cholesterol) levels were not related to SNP rs7903456 and therefore might not confound the association signal. To examine the potential multi-collinearity effects in our multivariate model, we have calculated the VIF (variance inflation factor) and R^2^ for these 6 clinical variables. The results are summarized in Supplemental Table S2. No signs of multi-collinearity can be identified, and therefore, these clinical variables could be included in the full model altogether. For the multivariate model, we have included age, gender, and these 6 clinical variables as covariates. A significant association signal was obtained for uric acid level only when rs7903456 was coded in the recessive mode (SNP was coded as 0 and 1 when there is either 0 or 1 minor allele and 2 minor alleles). After being adjusted by several clinical factors, the *P* value was 0.019 (*β* = 10.61). No multiple comparisons need to be addressed for the multivariate model because we only tested and presented one SNP. This significant hit was only identified for the uric acid level but not for disease status. The full results of the multivariate model for rs7903456 are summarized in Table 3.

**Table 3:** Full results of multivariate models fitted for rs7903456.

Phenotype	Genetic Codes	OR(Beta)	SE	Lower 95% CI	Upper 95% CI	STAT	*P*
hyperuricemia	Additive	1.19	0.12	0.93	1.52	1.41	0.16
	Dominant	1.37	0.16	0.99	1.88	1.91	0.06
	Recessive	0.97	0.28	0.56	1.68	−0.12	0.91
UA	Additive	2.38	1.95	−1.44	6.20	1.22	0.22
	Dominant	0.69	2.49	−4.18	5.57	0.28	0.78
	**Recessive**	**10.61**	**4.54**	**1.71**	**19.52**	**2.34**	**0.02**

CI: confidence interval.

### Bioinformatics analyses

**Figure 1:**
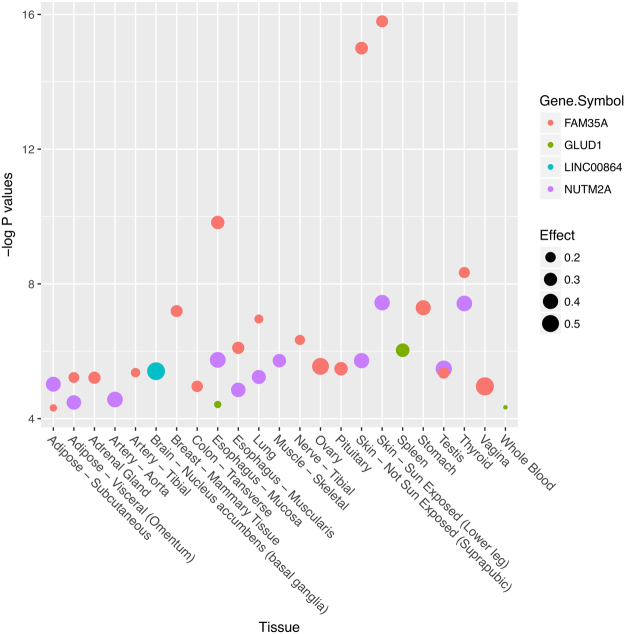
Significant effects of rs7903456 on gene expression in multiple human tissues.

We examined the potential effects of SNP rs7903456 on gene expression in multiple tissues by the GTEx (Genotype-Tissue Expression) database (Fig. 1). Rs7903456 had widespread significant effects on expression of multiple loci including *FAM35A, GLUD1, LINC00864* and *NUTM2A*, and these eQTL (Expression Quantitative Trait Loci) effects were identified in 23 human tissues. This finding indicated that SNP rs7903456 had significant biological function through affecting gene expression of *FAM35A*.

## Discussion

To the best of our knowledge, this study is the first to focus on the relationship between *FAM35A* genetic polymorphisms and hyperuricemia-related traits in the Chinese Han population. In this study, we have identified a significant SNP, rs7903456, to be associated with hyperuricemia and the related uric acid level. Although the significance of rs7903456 on the disease status disappeared after being adjusted by several potential confounders, its significance on the hyperuricemia-related quantitative trait was successfully attained. Our findings showed that the T allele of rs7903456 could increase the uric acid level by ~10 mmol/L on average after adjusting several biochemical and clinical variables. Our results were similar to the GWAS study of gout in the Japanese population published by Nakayama *et al*., although different phenotypes were investigated in the two studies. In the GWAS study, the T allele (known as the A allele in the original paper due to a different reference sequence) of rs7903456 would significantly increase the risk of gout by approximately 30%^12^. Our findings indicated that the effects of rs7903456 on gout might partly be mediated by its effect on the uric acid level because we now know that one of the most important risk factors for gout is hyperuricemia and its related biochemical variables.

*FAM35A* encodes a protein with 835 amino acids, and it is ubiquitously expressed in many organs and human tissues^17^. However, the biological function of *FAM35A* is still unclear. Nevertheless, combined with the results of our bioinformatics analyses using GTEx, one interesting point is worth noting. Our GTEx data showed that rs7903456 might have significant effects on four genes in multiple tissues. Therefore, it is valuable to move our focus from *FAM35A* to the other 3 genes. As shown in Fig. 1, the other three genes were *GLUD1*, *LINC00864* and *NUTM2A*. Similar to *FAM35A*, the biological function for *LINC00864* and *NUTM2A* are still unclear, but *GLUD1* is a gene with clearer biological functions. *GLUD1* encodes glutamate dehydrogenase, which plays an important role in human nitrogen metabolism. It is located at the mitochondrial matrix and catalyzes the oxidative deamination of glutamate into alpha-ketoglutarate and ammonia. Early family-based studies have linked this gene to hyperinsulinemic hypoglycemia and hyperammonemia^18–20^. Nevertheless, it is still too early to conclude anything with this vague bioinformatics evidence. More studies are needed in the future to 1) confirm the biological function of *FAM35A* and 2) investigate the potential association between *GLUD1* and hyperuricemia (and its related traits).

In our study, some limitations should be kept in the mind. One challenge of this study is to control confounding factors. As shown in Table 1 and Supplemental Fig. S2, most of the clinical variables we collected were associated with both hyperuricemia and our target SNP. Although we have adjusted these factors in our multivariate model, it is still possible that some underlying confounders have been missed. Some previous similar studies have included blood pressure measures as covariates in the multivariate model^10,11^, however, we did not collect these measures, so they were not adjusted in our model. This might introduce some confounding effects on our results. In addition, we did not apply any statistical techniques in addressing the potential population stratification in this genetic association study. As a candidate gene-based study, we simply did not have enough genetic markers to estimate the principle components for our sample. However, we have applied some criteria during the sample recruitment process to control the genetic heterogeneity of our sample by restricting their immigration history, and the Q-Q plot of our simple model did not show any signs of inflation for our tested markers. Finally, Population stratification is one of the major limitations to weaken the reliability of our significant results. However, we have tried our best to rule out the potential effects of population stratification by restricting the genetic background (through confining the immigration history of study subjects) during the sample collection^21,22^.

In sum, this study has identified a significant SNP located within *FAM35A* to be associated with hyperuricemia and the blood uric acid level, and after adjusting for multiple potential confounders, the association signal is still attained for the uric acid level. Our findings indicated that the previously identified effects of rs7903456 on gout in Japanese population might be partly mediated by its effect on the uric acid level. More studies in the future are needed to clarify the biological functions of *FAM35A* and *NUTM2A*. In addition, it might be worth diverting the focus from *FAM35A* to *GLUD1*, which is related to nitrogen metabolism since our bioinformatics analyses showed that rs7903456 was also associated with the expression of *GLUD1*.

## Electronic supplementary material

Supplemental Materials
