# Supplementary material for: Genetic association and functional analysis of rs7903456 in FAM35A gene and hyperuricemia: a population based study
Source: Sci Rep. 2018 Jun 25;8:9579. doi: 10.1038/s41598-018-27956-3 (PMC6018507; doi:10.1038/s41598-018-27956-3)
Supplement: Supplementary file 1 — Supplemental Materials [file 41598_2018_27956_MOESM1_ESM.docx]

***Title***: Genetic association and functional analysis of rs7903456 in *FAM35A* gene and hyperuricemia: a population based study

***Author names and affiliations***: Feng Yan ^1^, Peng Sun ^2^, Huishou Zhao ^3^, Changhai Zhao ^1^, Nana Zhang ^4^ and Yujie Dai ^1^

^1^ Department of Clinical Nutrition, Xijing Hospital, the Fourth Military Medical University, Xi'an, Shaanxi, China;

^2^ Department of Neurosurgery, Tangdu Hospital, the Fourth Military Medical University, Xi'an, Shaanxi, China;

^3^ Department of Cardiology, Xijing Hospital, the Fourth Military Medical University, Xi'an, Shaanxi, China;

^4^ Department of Endocrinology, Xijing Hospital, the Fourth Military Medical University, Xi'an, Shaanxi, China

***Corresponding Author***:

Feng Yan, M.D. & Ph.D., Department of Clinical Nutrition, Xijing Hospital, the Fourth Military Medical University, No.169, Changle West Road, Beilin District, Xi'an, Shaanxi, China, 710000.

Tel: 86-29- 84773413; Fax: 86-29- 84773413; E-mail: fyannutri@163.com

Supplemental Table S1. Basic information of the 31 selected SNPs.

| CHR | Position | SNP | Allele | FUNC | GENE | MAF | HARDY |
| --- | --- | --- | --- | --- | --- | --- | --- |
| 10 | 87096385 | rs67141022 | A/C | intron,near-gene-5 | *FAM35A* | 0.10 | 0.52 |
| 10 | 87103090 | rs181570058 | A/G | intron | *FAM35A* | 0.03 | 0.65 |
| 10 | 87113822 | rs148634296 | A/G | intron | *FAM35A* | 0.26 | 0.66 |
| 10 | 87120536 | rs186029096 | C/T | intron | *FAM35A* | 0.06 | 0.56 |
| 10 | 87126743 | rs7089418 | C/T | intron | *FAM35A* | 0.12 | 0.73 |
| 10 | 87129688 | rs200289839 | A/G | intron | *FAM35A* | 0.11 | 1.00 |
| 10 | 87140690 | rs375246821 | A/G | intron | *FAM35A* | 0.02 | 1.00 |
| 10 | 87147082 | rs1885931 | A/C | intron,near-gene-5 | *FAM35A* | 0.39 | 0.66 |
| 10 | 87147698 | rs10466230 | C/T | intron,near-gene-5 | *FAM35A* | 0.04 | 0.14 |
| 10 | 87151309 | rs544765887 | C/T | intron,near-gene-5 | *FAM35A* | 0.02 | 1.00 |
| 10 | 87151317 | rs377405832 | A/G | intron,near-gene-5 | *FAM35A* | 0.02 | 1.00 |
| 10 | 87151670 | rs138542761 | A/C | intron,missense | *FAM35A* | 0.06 | 0.17 |
| 10 | 87154671 | rs575094327 | G/T | intron | *FAM35A* | 0.04 | 0.33 |
| 10 | 87156665 | rs151327238 | A/G | intron | *FAM35A* | 0.05 | 0.42 |
| 10 | 87157420 | rs200718310 | C/T | intron | *FAM35A* | 0.07 | 0.70 |
| 10 | 87158872 | rs116955887 | A/G | intron | *FAM35A* | 0.09 | 0.67 |
| 10 | 87159562 | rs7903456 | C/T | intron | *FAM35A* | 0.29 | 0.25 |
| 10 | 87162364 | rs187235859 | A/G | intron | *FAM35A* | 0.07 | 0.47 |
| 10 | 87164678 | rs550312128 | A/T | intron | *FAM35A* | 0.07 | 0.38 |
| 10 | 87165772 | rs4933432 | A/T | intron | *FAM35A* | 0.39 | 0.69 |
| 10 | 87166777 | rs191015049 | A/G | intron | *FAM35A* | 0.07 | 1.00 |
| 10 | 87167593 | rs374457039 | C/G | intron | *FAM35A* | 0.06 | 0.19 |
| 10 | 87168299 | rs533233110 | A/G | intron,near-gene-3 | *FAM35A* | 0.05 | 1.00 |
| 10 | 87169165 | rs55892577 | C/G | intron | *FAM35A* | 0.10 | 0.14 |
| 10 | 87170492 | rs11202365 | A/T | intron,missense | *FAM35A* | 0.21 | 0.77 |
| 10 | 87170893 | rs368106548 | A/G | intron,missense | *FAM35A* | 0.04 | 0.31 |
| 10 | 87171219 | rs117800846 | A/T | intron | *FAM35A* | 0.07 | 0.25 |
| 10 | 87175350 | rs189796790 | C/T | intron | *FAM35A* | 0.02 | 0.63 |
| 10 | 87177555 | rs185147308 | G/T | intron | *FAM35A* | 0.03 | 0.40 |
| 10 | 87179590 | rs190379103 | A/T | intron | *FAM35A* | 0.04 | 0.17 |
| 10 | 87180141 | rs77534648 | C/T | missense | *FAM35A* | 0.08 | 1.00 |

HARDY, *P* values of Hardy-Weinberg equilibrium test.

Supplemental Table S2. VIF and R^2^ of the 6 clinical variables.

| Variables | VIF | R^2^ |
| --- | --- | --- |
| BMI | 1.54 | 0.3507 |
| TC | 1.04 | 0.0379 |
| Triglyceride | 1.36 | 0.2644 |
| BG | 1.15 | 0.1339 |
| UN | 1.15 | 0.1270 |
| Creatinine | 1.49 | 0.3289 |

BG, fasting glucose level; BMI, body mass index; TC, total cholesterol; UN, urea nitrogen; VIF, variance inflation factor

Supplemental Figure S1. Q-Q plot of association signal between 31 selected SNPs and hyperuricemia disease status.


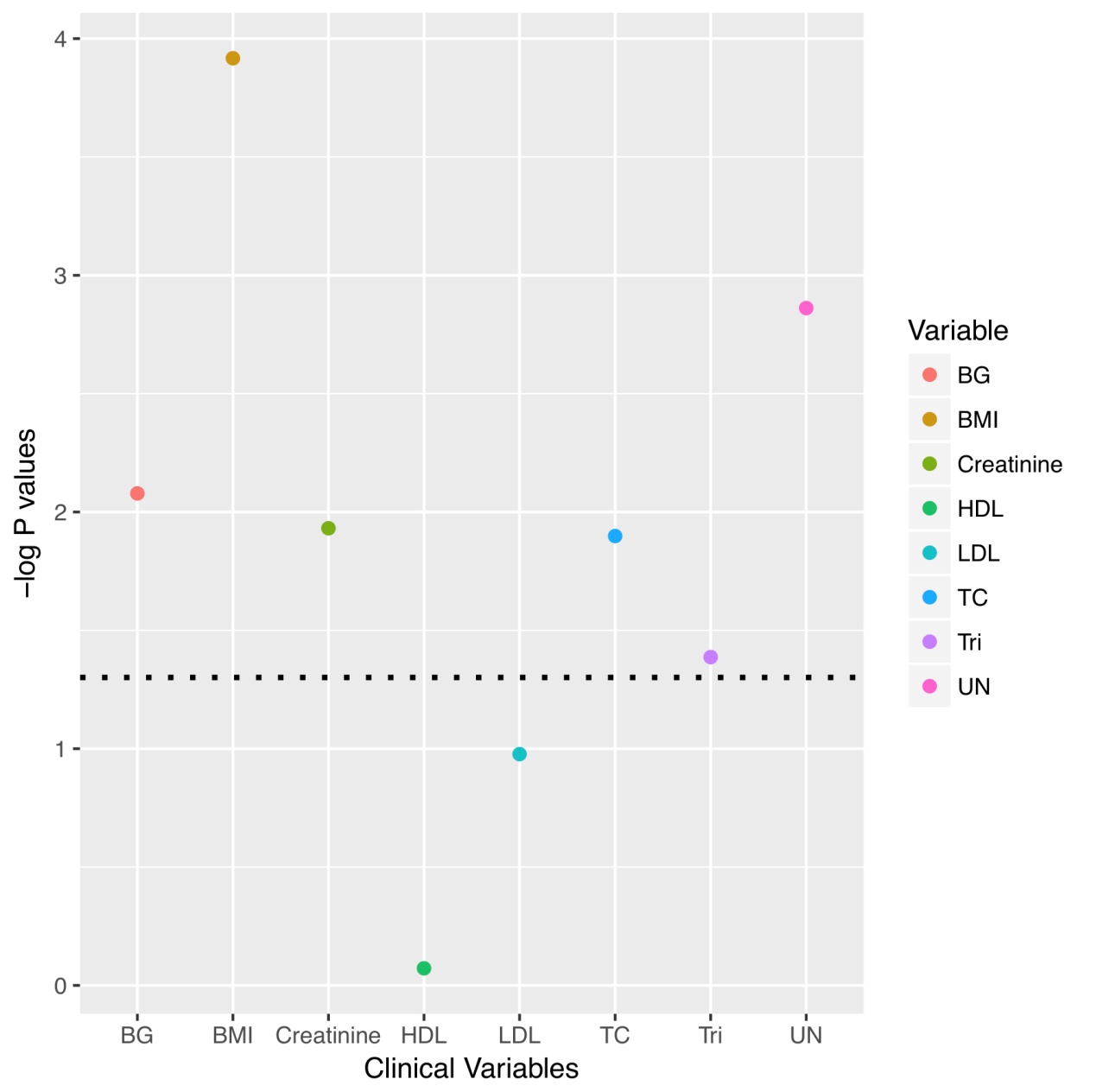


Supplemental Figure S2. Association signals adjusted by age and gender between SNP rs7903456 and 8 clinical variables. BG, fasting glucose level; BMI, body mass index; HDL, high-density lipoprotein; LDL, low-density lipoprotein; TC, total cholesterol; Tri, triglyceride; UN, urea nitrogen. The *P* value threshold was indicated by the dotted horizontal line in this plot.
